# Renal Changes and Apoptosis Caused By Subacute Exposure To Aroclor 1254 in Selenium-deficient and Selenium-supplemented Rats

**DOI:** 10.2478/aiht-2020-71-3360

**Published:** 2020-06-29

**Authors:** Naciye Dilara Zeybek, Ünzile Sur, Ofcan Oflaz, Pınar Erkekoğlu, Aylin Balcı, Gizem Özkemahlı, Ali Aşçı, Murat Kızılgün, Oğuz Han Edebal, Belma Koçer-Gümüşel

**Affiliations:** 1 Hacettepe University Faculty of Medicine, Department of Histology and Embryology, Ankara, Turkey; 2 Hacettepe University Faculty of Pharmacy, Department of Toxicology, Ankara, Turkey; 3 Atatürk University Faculty of Pharmacy, Department of Toxicology, Erzurum, Turkey; 4 Hacettepe University Faculty of Science, Department of Biology, Ankara, Turkey; 5 Erzincan Binali Yıldırım University Faculty of Pharmacy, Department of Toxicology, Erzincan, Turkey; 6 Dışkapı Children’s Health and Diseases, Haematology, and Oncology Training and Research Hospital, Ankara, Turkey; 7 Lokman Hekim University Faculty of Pharmacy, Department of Toxicology, Ankara, Turkey

**Keywords:** antioxidant enzymes, electron microscopy, histopathology, kidney, oxidative stress, polychlorinated biphenyls, Sprague-Dawley rats, TUNEL assay, ultrastructural changes, antioksidacijski enzimi, bubrezi, oksidacijski stres, poliklorirani bifenili, štakori Sprague-Dawley, TUNEL metoda

## Abstract

Aroclor 1254 (A1254), a mixture of polychlorinated biphenyls, exerts hepatic, renal, and reproductive toxicity in rodents. This study aimed to determine a protective role of selenium on histopathological changes, oxidative stress, and apoptosis caused by A1254 in rat kidney. It included a control group, which received regular diet containing 0.15 mg/kg Se (C), a Se-supplemented group (SeS) receiving 1 mg/kg Se, a Se-deficient group (SeD) receiving Se-deficient diet of ≤0.05 mg/kg Se, an A1254-treated group (A) receiving 10 mg/kg of Aroclor 1254 and regular diet, an A1254-treated group receiving Se-supplementation (ASeS), and an A1254-treated group receiving Se-deficient diet (ASeD). Treatments lasted 15 days. After 24 h of the last dose of A1254, the animals were decapitated under anaesthesia and their renal antioxidant enzyme activities, lipid peroxidation (LP), glutathione, protein oxidation, and total antioxidant capacity levels measured. Histopathological changes were evaluated by light and electron microscopy. Apoptosis was detected with the TUNEL assay. Kidney weights, CAT activities, and GSH levels decreased significantly in all A1254-treated groups. Renal atrophic changes and higher apoptotic cell counts were observed in the A and ASeD groups. Both groups also showed a significant drop in GPx1 activities (A – 34.92 % and ASeD – 86.46 %) and rise in LP (A – 30.45 % and ASeD – 20.44 %) vs control. In contrast, LP levels and apoptotic cell counts were significantly lower in the ASeS group vs the A group. Histopathological changes and renal apoptosis were particularly visible in the ASeD group. Our findings suggest that selenium supplementation provides partial protection against renal toxicity of Aroclor 1254.

## Abstract (HR)

Mješavina polikloriranih bifenila poznata pod nazivom aroklor 1254 (A1254) dokazano je toksična za jetru, bubrege i reprodukcijski sustav u glodavaca. Cilj ovoga istraživanja bio je na temelju histoloških promjena te parametara oksidacijskoga stresa i apoptoze utvrditi u kojoj mjeri selenij (Se) štiti bubrege od njegove toksičnosti. Istraživanje je obuhvatilo kontrolnu skupinu na normalnoj prehrani, koja je sadržavala 0,15 mg/kg Se, zatim skupinu čija je prehrana bila obogaćena selenijem (SeS) u dozi od 1 mg/kg, skupinu čija je prehrana bila osiromašena selenijem (SeD) (≤0.05 mg/ kg), skupinu koja je uz uobičajenu prehranu bila izložena arokloru 1254 (A) u dozi od 10 mg/kg tjelesne težine, skupinu koja je uz prehranu obogaćenu selenijem bila izložena arokloru 1254 (ASeS) i konačno skupinu koja je uz prehranu osiromašenu selenijem bila izložena arokloru 1254 (ASeD). Izloženost arokloru 1254 trajala je 15 dana. Životinje su 24 sata nakon primitka posljednje doze aroklora 1254 bile dekapitirane pod općom anestezijom te su im izmjerene vrijednosti bubrežnih enzimskih aktivnosti, lipidne peroksidacije (LP), glutationa (GSH), proteinske oksidacije i ukupnoga antioksidacijskoga kapaciteta. Histopatološke promjene utvrđene su pomoću svjetlosne i elektronske mikroskopije. Broj apoptotskih stanica utvrđen je TUNEL metodom. U svih skupina izloženih arokloru 1254 uočen je pad težine bubrega te aktivnosti katalaze i glutationa. U skupinama A i ASeD također je uočen povišen broj apoptotskih stanica i atrofija bubrežnoga tkiva, značajan pad aktivnosti GPX1 (u skupini A za 34,92 %, a u skupini ASeD za 86,46 %) te porast lipidnih peroksida (u skupini A za 30,45 %, a u skupini ASeD za 20,44 %) u odnosu na kontrolnu skupinu. Nasuprot skupini A, razine lipidnih peroksida i broj apoptotskih stanica bili su značajno niži u skupini ASeS, koja je primala hranu obogaćenu selenijem. Histopatološke promjene i apoptoza bubrežnih stanica osobito su se isticale u skupini ASeD na hrani osiromašenoj selenijem. Naši rezultati upućuju na to da nadomjesna primjena selenija pruža barem djelomičnu zaštitu od toksičnoga djelovanja aroklora 1254 na bubrege.

Polychlorinated biphenyls (PCBs) are toxic environmental contaminants with persistent, lipophilic, and strongly hydrophobic properties (1, 2). Aroclor 1254 (A1254) is a commercial mixture of PCBs used as a non-flammable heat transfer agent in electric capacitors, power transformers, vacuum pumps, and gas-transmission turbines (3). Its exposure route is through ingestion of contaminated food and water, inhalation of contaminated air, or through skin in contact with contaminated surfaces (4, 5). A1254 affects different systems, including the renal system, and these effects may be related to oxidative stress (6, 7, 8, 9, 10) caused by high levels of reactive oxygen species (ROS), which can damage the cellular ultrastructure and lead to cell death (necrosis, apoptosis, and autophagy) (11, 12). ROS also targets glycolipids, phospholipids, and cholesterol and induces lipid peroxidation, which, in turn, forms a series of cell membrane-damaging products, such as malondialdehyde, and may be lethal (13). In addition, lipid oxidation may lead to protein oxidation and therefore to DNA damage (13, 14).

Although several PCB congeners have been banned in different countries, humans are still exposed to them due to their persistence in the environment. The Joint Food and Agriculture Organization (FAO) and World Health Organisation (WHO) Expert Committee on Food Additives (JECFA) has estimated that dietary PCB intake in adults is 0.005–2 μg/kg bw/day, while in breast-fed infants it is as high as 2–12 μg/kg bw/day (15). A number of studies (9, 10, 16, 17, 18, 19, 20, 21) suggest that PCB congeners including Aroclor1254 cause hepatotoxicity, carcinogenicity, neurotoxicity, reproductive disorders, endocrine disruption, teratogenicity, and numerous other biochemical alterations, but only a few studies have investigated renal toxicity of A1254 and showed histological changes (under light microscopy) or genotoxic effect (6, 22, 23). As the kidney contains high selenium (Se) levels in both rodents and humans (24, 25) and as Se has proven antioxidant and antiapoptotic properties (26, 27, 28, 29), the aim of this study was to look deeper and evaluate ultrastructural histopathological changes caused by A1254 and related total antioxidant levels, lipid peroxidation, and apoptotic cell death and for the first time to determine the protective effects of selenocompounds against A1254 in the kidney.

## Materials and methods

### Chemicals and kits

All chemicals were obtained from Sigma-Aldrich (St. Louis, MO, USA). A1254 (analytical standard) was obtained from the German, Manheim branch of Sigma-Aldrich. Assay kits for measuring total antioxidant capacity (TAOC), thiobarbituric acid reactive substances (TBARS), protein carbonyls (colorimetric), glutathione (GSH), superoxide dismutase (SOD), catalase (CAT), and glutathione peroxidase 1 (GPx1) were supplied by Cayman Chemical Company (Ann Arbor, MI, USA).

### Animals and experimental design

The study included 36 three-week-old male Sprague-Dawley rats purchased from the Experimental Animals Production Centre of Hacettepe University (Ankara, Turkey). All experimental procedures and animal use were approved by the Animals Ethics Committee of Hacettepe University. The experiments followed the regulations of the Ministry of Food, Agriculture, and Livestock and Hacettepe University Animal Ethics Committee (30, 31).

The animals had free access to food and water during the five-week experiment and were randomly divided in six groups of six. The first, control group was receiving a regular rat chow containing 0.15 mg/kg of Se. The second was receiving rat chow supplemented with 1 mg/kg of Se (SeS group). The third group was receiving Se-deficient rat chow (≤0.05 mg/kg of Se) (SeD group). The fourth group was receiving regular rat chow with 0.15 mg/kg of Se and 10 mg/kg of Aroclor 1254 by gavage for the last 15 days of feeding (A group). The fifth group was receiving Se-supplemented rat chow (1 mg/kg Se) and 10 mg/kg of A1254 by gavage for the last 15 days of feeding (ASeS group). The sixth group was receiving Se-deficient rat chow (≤0.05 mg/kg of Se) and 10 mg/kg of A1254 by gavage for the last 15 days of feeding (ASeD group).

The A1254 dose of 10 mg/kg/day corresponded to 1 % of median lethal oral dose (LD_50_) of 1010 mg/kg (5) and was diluted in 1 mL of corn oil as vehicle. Corn oil was also given by gavage to the control, SeS, and SeD groups over the last 15 days of feeding.

Control, Se-deficient, and Se-supplemented diets were obtained from the Scientific Animal Food and Engineering (SAFE) laboratories (Augy, France) and contained sodium selenite as a source of Se. Se doses in both supplemented and deficient chow were based on recent studies (32, 33, 34), Sundae (34) in particular, which has showed that 0.05 mg/kg bw of Se ensures 50 % of normal GPx1 activity, while 0.15 mg/kg ensures normal activity. Diet containing 1 mg/kg was evidenced to provide supraphysiological doses of Se (23, 34).

The animals were decapitated under total anaesthesia 24 h after having received the last dose of A1254, and their kidneys removed and weighed. The right kidney was prepared for histopathological analysis and apoptotic cell count, whereas the left kidney was frozen at -80 °C until tissue homogenisation for oxidant/antioxidant parameter analysis.

### Histopathological analysis

Samples of one half of the right kidney were fixed in 2.5 % glutaraldehyde solution for 4 h and post-fixed in 1 % osmium-tetroxide solution in 0.1 mol/L phosphate-buffer for 1 h. They were then dehydrated in a graded series of ethanol and embedded in Araldite/Epon812 (Cat. No. 13940, EMS, PA, USA). After heat polymerisation, the samples were cut in 1 μm thick sections, stained with methylene blue-azure II, and examined at 400x magnification under a light microscope (Leica DM6000B, Wetzlar, Germany) with a DC490 digital camera (Leica). Ultrathin (70 nm thick) sections cut with an ultramicrotome (Leica ultracut R) were double-stained with uranyl acetate and lead citrate (Leica EM AC20), examined under a JEOL-JEM 1400 electron microscope, and photographed with a CCD camera (Gatan, Pleasanton, CA, USA).

### Apoptotic cell count with the TUNEL assay

Samples of the other half of the right kidney were fixed in 10 % formalin solution, dehydrated, and embedded in paraffin. Their sections were then incubated with 0.1 % Triton X-100 in 0.1 % sodium citrate for permeabilisation at 4 °C for 8 min, washed, and incubated with a TUNEL reagent at 37 °C for 1 h and washed again. They were then treated with a converter reagent at 37 °C for 30 min. After washing, the sections were incubated with Fast Red substrate solution for 10 min. Negative control sections were incubated with a reaction mixture without terminal deoxynucleotidyl transferase (TdT). TUNEL-positive apoptotic cells (red-labelled) were counted in ten random fields of cortex and medulla with a light microscope at 400x magnification (one sample per animal). The count is given as the mean number of TUNEL-positive cells per field per group.

### Kidney homogenate preparation

Left kidney homogenates were prepared in ice-cold buffer containing Tris (10 mmol/L, adjusted to pH 7.4) and protease inhibitors to obtain 10 % (w/v) whole homogenate. After different stages of centrifugation (1500 x *g*) at 4 °C, the supernatant was used to measure TAOC, malondialdehyde (MDA) concentration, and protein oxidation.

### Determination of antioxidant enzyme activities

For all spectrophotometric measurements we used SpectraMax M2 (Molecular Devices, CA, USA). Renal GPx activity was measured indirectly through coupled reaction with glutathione reductase (GR). Absorbance was measured at 340 nm at one-minute intervals, and GPx1 activity expressed as nmol/mg of protein per min.

CAT activity was measured with a commercial spectrophotometric kit, which utilises the peroxidative function of CAT for determination of enzyme activity. The absorbance values were measured at 540 nm, and CAT activity was expressed as nmol/mg of protein per min.

SOD activity was measured with a commercial colorimetric kit with a radical detector [i.e. 2-(4-iodophenyl)-3-(4-nitrophenyl)-5-(2,4-disulfophenyl)-2H-tetrazolium (WST-1), monosodium salt] that produces a water-soluble formazan dye upon reduction with a superoxide anion. One unit of SOD is defined as the amount of enzyme needed to exhibit 50 % dismutation of the superoxide radical. SOD activity was expressed as U/mg of protein.

### TAOC determination

This assay uses the ability of intracellular antioxidants to inhibit the oxidation of 2,2’-azino-di-(3-ethylbenzthiazoline sulphonate) (ABTS) to ABTS^+^ by metmyoglobin at 405 nm. The capacity of these antioxidants to inhibit ABTS+ oxidation was compared to the same ability of Trolox. TAOC levels were expressed as mmol/L of Trolox equivalents per mg protein.

### Determination of lipid peroxidation

Renal lipid peroxidation was quantified with a TBARS assay kit, which measures the concentration of MDA as an important indicator of lipid peroxidation. MDA forms a complex with thiobarbituric acid (TBA) at 90–100 °C under acidic conditions. The colour intensity of the MDA-TBA complex was measured at 540 nm spectrophotometrically, and MDA concentration calculated based on MDA standards (0, 0.5, 5, 10, 20, 30, and 50 μmol/L) and expressed as μmol/L.

### Determination of protein oxidation

The level of carbonyl groups as an indicator of protein oxidation was determined by derivatising them with 2,4 dinitrophenylhydrazine (DNPH) to form stable dinitrophenyl (DNP) hydrazone adducts, whose levels are proportional to the carbonyls present and are measured at detected at 360 nm. Carbonyl levels were expressed as nmol/mg protein.

### Determination of total GSH

Kidney GSH levels were measured with a commercial kit based on the reaction of its sulphhydryl group with 5,5’-dithio-bis-(2-nitrobenzoic acid) (DTNB) to produce a yellow-coloured compound of GSH and 5-thio-2-nitrobenzoic acid (TNB). The absorbance values of the samples were measured at 414 nm. The results were expressed as nmol/mg of protein.

### Total protein determination

Protein content of the kidney samples was determined with a protein assay kit bicinchoninic acid as described elsewhere (35).

### Statistical analysis

The obtained data were analysed with one-way analysis of variance (ANOVA), followed by the Mann-Whitney U test. We used the Statistical Package for Social Sciences Program (SPSS, Chicago, IL, USA) version 17.0. Statistical significance was set at p<0.05. The results were given as mean ± standard error of the mean (SEM).

## Results

### Changes in kidney weight

**Figure 1:**
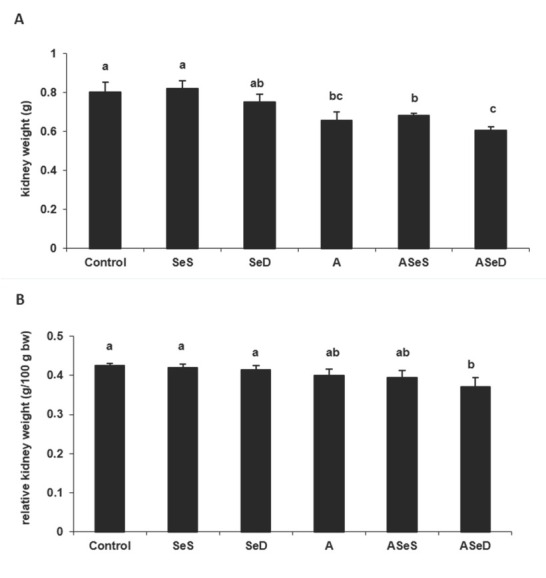
Kidney absolute and relative kidney weights in the experimental groups. Bars represent means ±SEM. Bars with different letters differ significantly from each other (p<0.05). A – absolute kidney weight B– relative kidney weight

In all A1254-exposed groups, kidney weights decreased significantly compared to control (Figure 1), whereas relative kidney weights were significantly lower only in the ASeD group (12.65 %).

### Kidney tissue light microscopy

**Figure 2:**
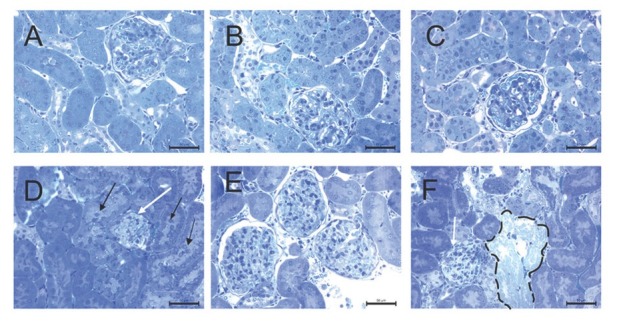
Histopathological analysis of the renal cortex in the experimental groups. Narrowing in the Bowman’s space, renal corpuscular atrophy (white arrow), peritubular vascular congestion, oedema, desquamation of epithelial cells (black arrow) in the A group and necrotic tubules (dashed area) in the ASeD group. A– control; B– Se-supplemented (SeS) group; C– Se-deficient (SeD) group; D – A1254-treated (A) group; E– Se-supplemented A1254-treated (ASeS) group; F– Se-deficient A1254-treated (ASeD) group

**Figure 3:**
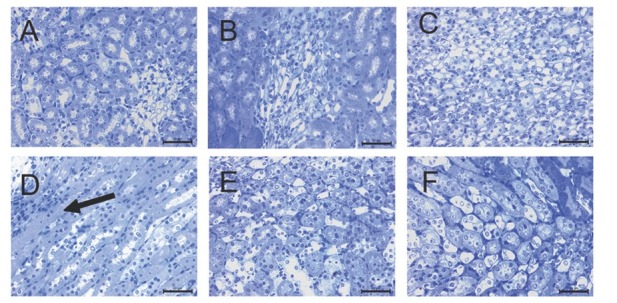
Histopathological analysis of the renal medulla in the experimental groups. Denudation of renal tubular cells (black arrow) in the A group. A– control; B– Se-supplemented (SeS) group; C– Se-deficient (SeD) group; D– A1254-treated (A) group; E – Se-supplemented A1254-treated (ASeS) group; F– Se-deficient A1254-treated (ASeD) group

Control and the SeS samples showed normal structure of the renal cortex and the medulla. SeD samples showed desquamation of epithelial cells in several renal tubules. A and ASeD samples, however, showed narrowing of the Bowman’s space, renal corpuscular atrophy, vascular congestion, and oedema. Epithelial cells of the tubules were swollen, and desquamated in the cortex and medulla. In addition, ASeD samples showed necrosis and hyaline cast in the tubules. ASeS samples showed peritubular vascular congestion both in the cortex and medulla, but without corpuscular atrophy (Figures 2 and 3).

### Kidney tissue electron microscopy

**Figure 4:**
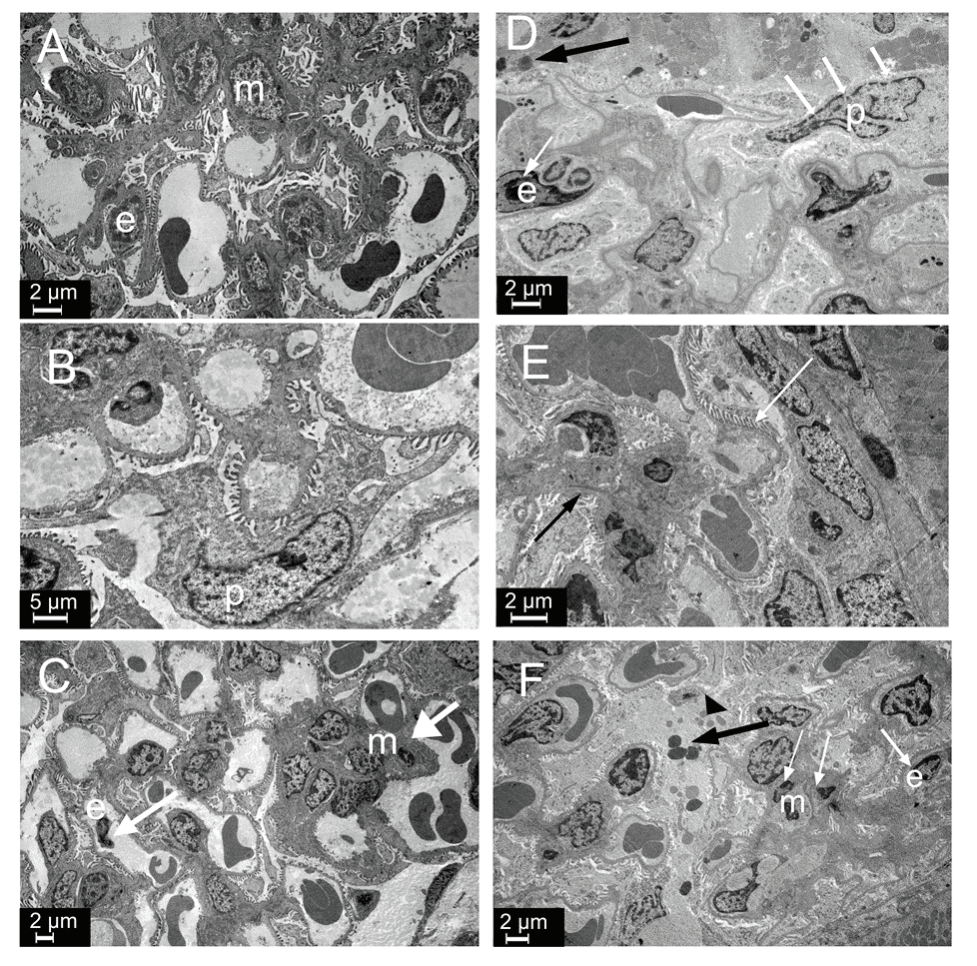
Ultrastructure of glomeruli in the experimental groups. Apoptotic (white arrow), endothelial (e), and mesangial (m) cells in the SeD group. Lipid droplets (arrow head) and lysosomes (black arrow) in the cytoplasm of podocytes and apoptotic cells (white arrow) in the A and ASeD groups. Regular pedicels (white arrow), effacement of the pedicels (black arrow) in the ASeS group. p – podocyte; m – mesangial cell; e – endothelial cell; A – control; B – Se-supplemented (SeS) group; C – Se-deficient (SeD) group; D – A1254-treated (A) group; E – Se-supplemented A1254-treated (ASeS) group; F – Se-deficient A1254-treated (ASeD) group

The glomerular ultrastructure of control, SeS, and SeD samples was normal, showing open urinary spaces and regular pedicels (Figures 4A, 4B, 4C), except that SeD samples had a few endothelial, mesangial, and visceral epithelial apoptotic cells with condensed nuclei (Figure 4C). In contrast, A1254 treatment caused degeneration of the glomerulus and tubules. Urinary space in A samples was filled with cytoplasmic extensions of podocytes, whose cytoplasm, in turn, showed lipid droplets and lysosomes. Some podocytes showed typical signs of apoptosis, such as chromatin condensation. In some areas, pedicels were effaced. A samples also showed capillaries filled with electron lucent material and apoptotic endothelial cells (Figure 4D). In ASeS samples pedicels were regular and the distance between them was equal, but some were effaced (Figure 4E). Lipid droplets and lysosomes were prominent in podocyte cytoplasm of ASeD samples, and cytoplasmic extensions were filling the urinary space. These samples also showed apoptotic mesangial and endothelial cells (Figure 4F).

**Figure 5:**
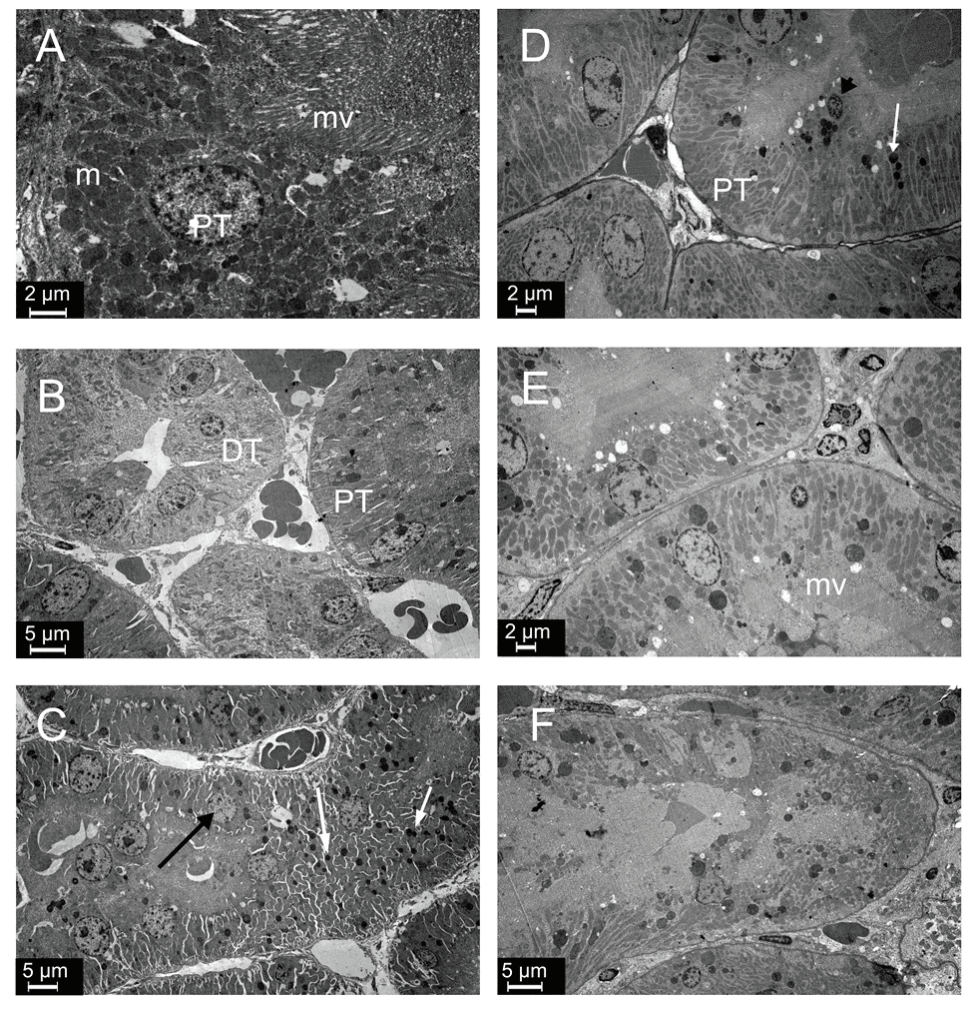
Ultrastructure of tubules in experimental groups. Lysosomes (white arrow) and karyolitic nuclei (black arrow) in the SeD group. Lysosomes (white arrow), apoptotic cells (black arrow head), and loss of microvilli in the A1254 group. Degeneration of tubular epithelial cells in the ASeD group. PT – proximal tubule; DT – distal tubule; m – mitochondria; mv – microvilli; A– control; B – Se-supplemented (SeS) group; C – Se-deficient (SeD) group; D – A1254-treated (A) group; E – Se-supplemented A1254-treated (ASeS) group; F – Se-deficient A1254-treated (ASeD) group

Figure 5 compares tubular ultrastructures between the groups. As expected, proximal and distal tubules showed normal ultrastructure in control and SeS samples (Figures 5A and 5B). SeD samples showed numerous lysosomes with electron-dense areas and karyolitic nuclei in the epithelium of proximal tubules (Figure 5C). A samples showed electron lucent material in spaces between the basal and lateral folds. The nuclei were karyolitic, and tubular epithelial cells showed lysosomes in the cytoplasm. The crista of mitochondria could not be distinguished. Apical microvilli in the proximal tubules were short, flat, and also indistinct in some areas (Figure 5D). ASeS samples showed microvilli in the apical part of proximal tubule epithelial cells (Figure 5E). ASeD samples showed some tubular necrosis. Epithelial cells lost integrity and were sloughed to the lumen in some areas. Collagen fibres were abundant in the interstitium, which also showed oedema (Figure 5F).

### Apoptotic cell counts

**Figure 6:**
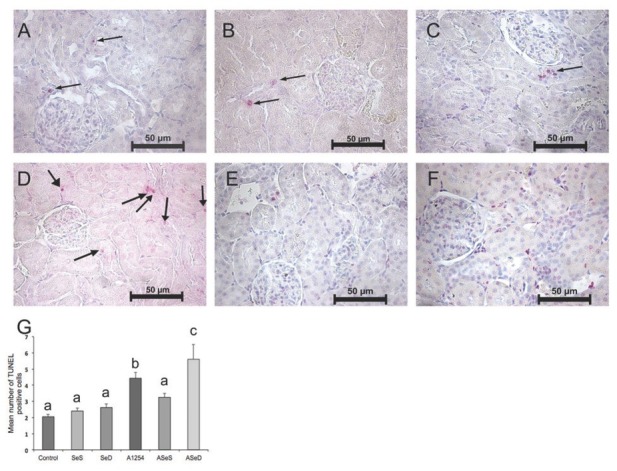
TUNEL assay of the renal cortex and apoptotic cell counts in the experimental groups. Positive nuclei for apoptosis are labeled pink. Arrows indicate TUNEL-positive (apoptotic) cells (400x magnification). Bars represent means ±SEM. Bars with different letters differ significantly from each other (p<0.05). A– control; B – Se-supplemented (SeS) group; C – Se-deficient (SeD) group; D – A1254-treated (A) group; E– Se-supplemented A1254-treated (ASeS) group; F – Se-deficient A1254-treated (ASeD) group

**Figure 7:**
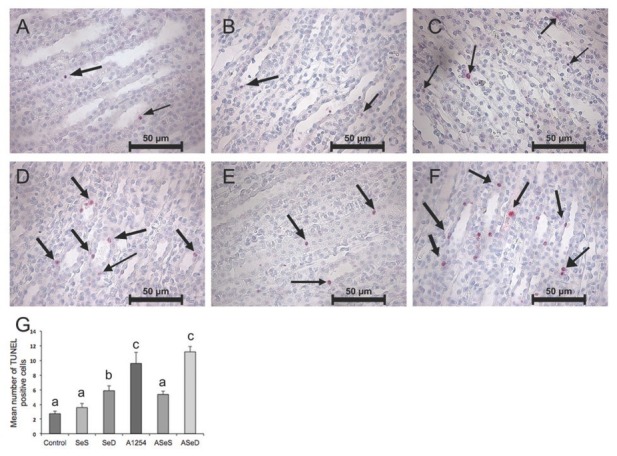
TUNEL assay of the renal medulla in experimental groups. Positive nuclei for apoptosis are labeled pink by the chromogenic reaction of Fast Red substrate by alkaline phosphatase (400x magnification).Arrows indicate TUNEL-positive (apoptotic) cells. Bars represent means ±SEM. Bars with different letters differ significantly from each other (p<0.05). A– control; B – Se-supplemented (SeS) group; C – Se-deficient (SeD) group; D – A1254-treated (A) group; E – Se-supplemented A1254-treated (ASeS) group; F – Se-deficient A1254-treated (ASeD) group

Figures 6 and 7 show the apoptotic (TUNEL-positive) cells and their counts in the cortex and medulla. Kidney cortex cell counts did not differ between the control, SeS, and SeD groups, but Se deficiency significantly contributed to medullar apoptosis compared to control. A1254 caused significant increases in apoptotic cell counts in both the cortex (almost 2-fold) and the medulla (3.5-fold) compared to control. Judging by significantly lower apoptotic cell counts in either the cortex and medulla of the ASeS group compared to the A group, Se treatment was highly beneficial against A1254 induced renal apoptotic cell death in rats.

### Antioxidant enzyme activities

**Figure 8:**
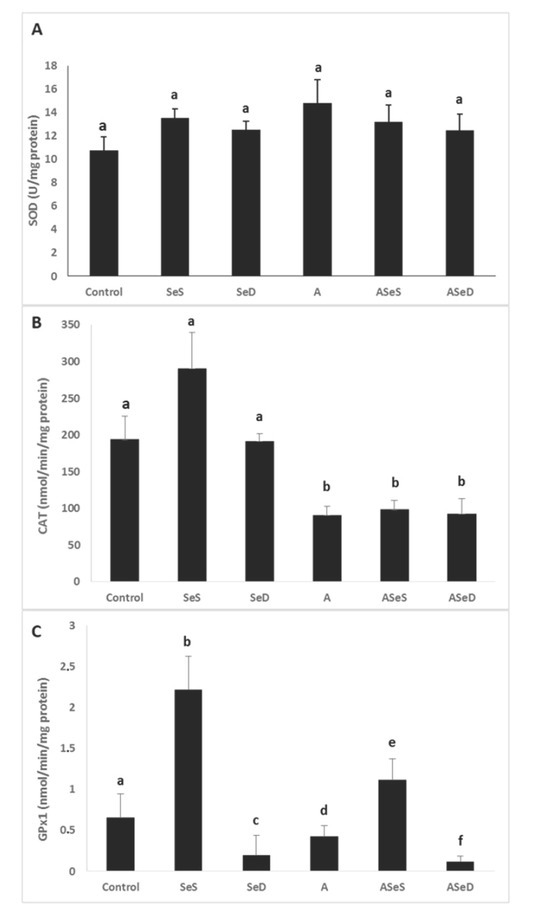
Antioxidant enzyme activities in the experimental groups. Bars represent means ±SEM. Bars with different letters differ significantly from each other (p<0.05). A – SOD activities; B – CAT activities; C – GPx1 activities SOD – superoxide dismutase; CAT – catalase; GPx1 – glutathione peroxidase; Se-supplemented (SeS) group; Se-deficient (SeD) group; A1254-treated (A) group; Se-supplemented A1254-treated (ASeS) group; Se-deficient A1254-treated (ASeD) group

The activities of SOD, CAT, and GPx1 are given in Figure 8. SOD activities did not significantly change in any of the groups, although they were 37.49 % higher in the A group than control. CAT activities significantly dropped in all A1254-exposed groups (52.96 % in the A, 48.98 % in the ASeS, and 52.05 % in the ASeD group; p<0.05). GPx1 activity was significantly higher in the SeS group than control group (3.38-fold increase), but markedly lower in the SeD group (70.41 %). It also significantly dropped in the A and ASeD groups (34.92 % and 86.46 %, respectively; p<0.05) compared to control.

### TAOC, lipid peroxidation, protein oxidation, and total GSH findings

**Figure 9:**
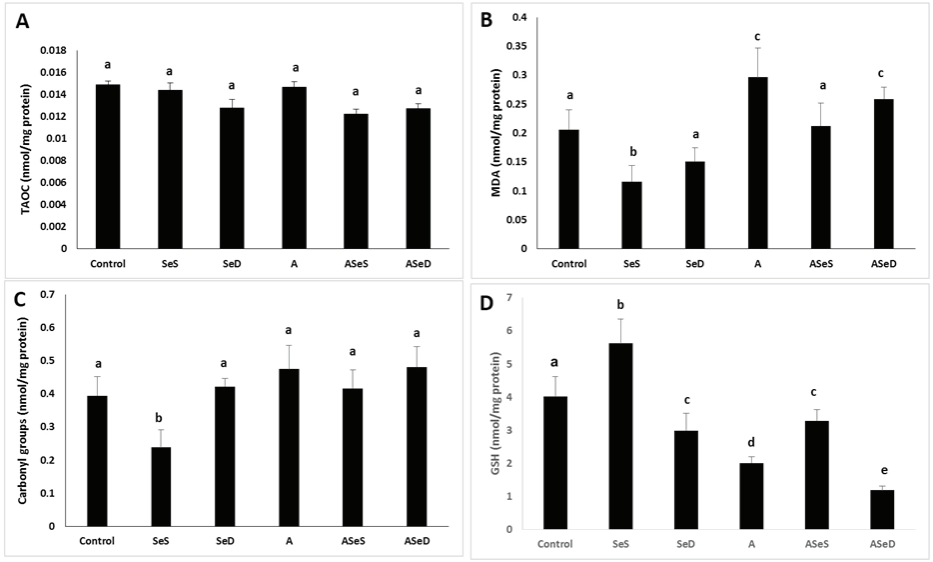
TAOC (A), MDA (B), carbonyl group (C) and total GSH (D) levels in the study groups. Bars represent means ±SEM. Bars with different letters differ significantly from each other (p<0.05). MDA – malondialdehyde; TAOC – total antioxidant capacity; Se-supplemented (SeS) group; Se-deficient (SeD) group; A1254-treated (A) group; Se-supplemented A1254-treated (ASeS) group; Se-deficient A1254-treated (ASeD) group

We did not observe any significant changes in renal TAOC and carbonyl levels in the A1254-exposed groups, but lipid peroxidation was significantly higher in the A and ASeD groups vs control (30.45 % and 20.44 %, respectively; p<0.05). In the SeS group, MDA and carbonyl levels were significantly lower vs control (43.59 % and 65.64 %, respectively) and all other study groups (p<0.05) (Figure 9).

The SeS group also had significantly higher total GSH vs control (39.80 %), but in all other study groups it was significantly lower (25.62 % in the SeD, 50 % in the A, 18.15 % in the ASeS, and 70.39 % in the ASeD group). However, Se supplementation provided significant increases (63.68 %) in total GSH levels compared to the A group. Se deficiency in A1254-exposed rats, in contrast, led to significantly lower GSH levels compared to A1254-exposed animals with normal selenium intake (A group) (36.84 %, p<0.05).

## Discussion

Our kidney weight findings confirm earlier reports that A1254 exposure decreases absolute kidney weight (6). However, Se supplementation in our study did not reverse this weight loss at all.

Our histopathological findings coincide with those reported by Kutlu et al. (22). Pedicel effacement, closure of the urinary space, and changes in the apical and basal surface of the tubular epithelium point to damaged glomerular filtration and damaged tubular absorption due to cell damage evidenced by ultrastructural findings of karyolitic nuclei in proximal tubules.

Our findings of numerous enlarged lysosomes that contained electron-dense areas in the Se-deficient group confirm the loss of the protective role of Se in the functioning of lysosomes and DNA integrity. Low Se levels have already been associated with kidney disease (36) and oxidative stress caused by different environmental chemicals (37). Se deficiency in our A1254-exposed animals resulted in areas with tubule necrosis and increased apoptotic cell counts in both cortex and medulla. Se supplementation, in turn, partly countered these damaging effects of A1254. These protective effects of Se against apoptosis confirm earlier studies involving a variety of toxic substances, including PCBs (24, 32, 38).

These histopathological and ultrastructural findings are in line with the lower renal CAT, GPx1, and total GSH levels and significantly higher lipid peroxidation in the A1254-exposed groups, Se-deficient in particular. They are also in line with earlier reports (6, 10, 39, 40). However, we observed no significant changes in TAOC and protein oxidation levels. Se deficiency in A1254-exposed rats did not further increase MDA levels, whereas Se supplementation was protective against lipid peroxidation caused by A1254.

Overall, our findings suggest that Se provides at least partial protection to the kidney against A1254, most likely through the induction (transcription and expression) of GPx1 as one of the most important antioxidant enzymes in the body (34). Several other mechanisms behind Se protection against environmental chemicals might be involved, but their role remains to be studied in the future.

This study has some limitations. It only shows changes in male rats and does not establish if there are sex-specific renal effects of A1254. In addition, we could have applied higher and lower doses of A1254 to rats to see if there are meaningful differences. We also could have used different age groups of animals to see if there are age-related effects of A1254.

## Conclusion

Despite these limitations, however, our study has shed more light on the protective effects of Se supplementation against the damaging effects of A1254 in rat kidney. Obviously, they were only partial, especially when it came to the ultrastructural damage caused by A1254. More mechanistic *in vitro* and *in vivo* studies are needed to show the interaction between kidney and other PCB congeners.
